# Prediction of 1-year mortality of patients treated for more than 72 hours in an ICU

**DOI:** 10.1186/cc13241

**Published:** 2014-03-17

**Authors:** S Steenbergen, S Rijkenberg, H Endeman

**Affiliations:** 1 Onze Lieve Vrouwe Gasthuis, Amsterdam, the Netherlands

## Introduction

ICU or hospital mortality rates have been reported as the endpoint of ICU therapy for many years. The aim of this study was to determine the 1-year mortality after discharge from the ICU in patients who were treated in the ICU for more than 72 hours and to identify predictors for 1-year mortality.

## Methods

This study was conducted in a 20-bed mixed ICU of a teaching hospital. The study sample was extracted from a dataset of all ICU patients treated for more than 72 hours between 1 January 2007 and 1 October 2012. Demographic characteristics and clinical characteristics at admission and during the ICU stay were collected. Characteristics of patients alive 1 year after ICU discharge were compared with patients who died within the first year after ICU discharge. Descriptive statistics were calculated. Multivariate analysis of 1-year mortality was performed using a logistic regression model with backward elimination. Survival was analysed by the Kaplan-Meier method using the time interval from day of ICU discharge until death.

## Results

During the study period, 740 patients were treated for more than 72 hours in the ICU. The ICU mortality was 106/740 (14%). The data of 617 ICU survivors were further analysed (17 patients were lost to follow up). One-year mortality was 175/617 (28%), of which 85/175 (49%) patients died during hospital stay after ICU discharge. Independent predicting factors of 1-year mortality were: age at ICU admission (OR: 1.03; 95% CI: 1.01 to 1.05), APACHE IV predicted mortality score (OR: 1.02; 95% CI: 1.02 to 1.03), number of comorbidities (one or two co morbidities OR: 2.14; 95% CI: 1.42 to 3.23) (>3 comorbidities OR: 2.56; 95% CI: 1.16 to 5.62), readmission after ICU discharge within the same period of hospital stay (OR: 1.98; 95% CI: 1.13 to 3.46) and the diagnosis at admission (cardiovascular OR: 4.31; 95% CI: 1.73 to 10.76) (sepsis OR: 2.67; 95% CI: 1.50 to 4.77).

## Conclusion

Of patients being treated for more than 72 hours in the ICU, 28% died within 1 year after ICU discharge. One-half of them within the hospital stay after ICU discharge. High age at ICU admission, high APACHE IV predicted mortality score, high number of comorbidities, readmission and an admission diagnosis within the categories 'cardiovascular' and 'sepsis' are associated with an increased 1-year mortality after ICU discharge in this population. The burden of patients dying after ICU discharge underlines the necessity for clear ICU discharge criteria and post-ICU care.

